# Implementation of an Internet-Based Acceptance and Commitment Therapy for Promoting Mental Health Among Migrant Live-in Caregivers in Canada: Protocol

**DOI:** 10.2196/31211

**Published:** 2021-09-13

**Authors:** Kenneth Po-Lun Fung, Mandana Vahabi, Masoomeh Moosapoor, Abdolreza Akbarian, Jenny Jing-Wen Liu, Josephine Pui-Hing Wong

**Affiliations:** 1 Department of Psychiatry Faculty of Medicine University of Toronto Toronto, ON Canada; 2 Krembil Research Institute Toronto Western Hospital University Health Network Toronto, ON Canada; 3 Daphne Cockwell School of Nursing Ryerson University Toronto, ON Canada; 4 Institute for Clinical Evaluative Sciences Toronto, ON Canada; 5 Dalla Lana School of Public Health University of Toronto Toronto, ON Canada

**Keywords:** migrant live-in caregiver, women, mental health, acceptance commitment therapy, resiliency, empowerment

## Abstract

**Background:**

Psychological distress, isolation, feelings of powerlessness, and limited social support are realities faced by temporary migrant live-in caregivers in Canada. Furthermore, they experience multiple barriers in accessing mental health services due to their long work hours, limited knowledge of health resources, precarious employment, and immigration status.

**Objective:**

The Women Empowerment - Caregiver Acceptance & Resilience E-Learning (WE2CARE) project is a pilot intervention research project that aims to promote the mental well-being and resiliency of migrant live-in caregivers. The objectives include exploring the effectiveness of this program in achieving the following: (1) reducing psychological distress (depression, anxiety, and stress); (2) promoting committed actions of self-care; and (3) building mutual support social networks. Further, participants’ satisfaction with the intervention and their perceived barriers to and facilitators of practicing the self-care strategies embedded in WE2CARE will be examined.

**Methods:**

A total of 36 live-in caregivers residing in the Greater Toronto Area will be recruited and randomly assigned to either the intervention or waitlist control group. The intervention group will receive a 6-week web-based psychosocial intervention that will be based on Acceptance and Commitment Therapy (ACT). Standardized self-reported surveys will be administered online preintervention, postintervention, and at 6 weeks postintervention to assess mental distress (Depression, Anxiety and Stress Scale), psychological flexibility (Acceptance and Action Questionnaire), mindfulness (Cognitive and Affective Mindfulness Scale – Revised), and resilience (Multi-System Model of Resilience Inventory). In addition, two focus groups will be held with a subset of participants to explore their feedback on the utility of the WE2CARE program.

**Results:**

WE2CARE was funded in January 2019 for a year. The protocol was approved by the research ethics boards of Ryerson University (REB 2019-036) and the University of Toronto (RIS37623) in February and May 2019, respectively. Data collection started upon ethics approval and was completed by May 2020. A total of 29 caregivers completed the study and 20 participated in the focus groups. Data analyses are in progress and results will be published in 2021.

**Conclusions:**

WE2CARE could be a promising approach to reducing stress, promoting resilience, and providing a virtual space for peer emotional support and collaborative learning among socially isolated and marginalized women. The results of this pilot study will inform the adaptation of an ACT-based psychological intervention for online delivery and determine its utility in promoting mental health among disadvantaged and vulnerable populations.

**International Registered Report Identifier (IRRID):**

DERR1-10.2196/31211

## Introduction

The Canadian Temporary Foreign Worker Program engages workers in sectors such as agriculture, petroleum, home caregiving, and other low-skilled occupations [1-3]. Temporary foreign workers (TFWs) are restricted to work only for the employers designated in their work permits. Most TFWs earn low wages, work long hours without any overtime compensation, live in substandard conditions, and have limited to no access to health care services or employment insurance despite paying into these programs [4-7]. The Caregiver Program, previously known as the Live-in Caregiver Program, is a stream of the Temporary Foreign Worker Program, which engages workers in in-home caregiving [1,8,9]. Despite Caregiver Program reforms in November 2014, which provided caregivers with the option of living outside of their employers’ homes, most caregivers continue to live with their employer due to low wages, inability to afford to live independently, and precarious work permits [5,6,10]. Most caregivers are racialized women from lower- or middle-income countries like the Philippines [2,11]. Live-in caregivers are one of the most vulnerable TFW groups because they live and work in private households where government surveillance remains nonexistent, employees’ privacy is gravely undermined, seclusion is often imposed, and unionization is unattainable [4,12]. TFWs are often the primary source of income for their families in their home country, sending back regular remittances and being compelled to tolerate substandard working and living conditions [4,6]. Although participants in this program are eligible to apply for permanent residency for themselves and their immediate families after 24 months of service, they are not allowed to bring their families to Canada until they become permanent residents, which could take 4 to 10 years [1,8]. In other words, family separation is one of the main requirements for receiving their work permit.

A limited but valuable body of literature examined the mental health of migrant live-in caregivers in Canada [13-16]. These studies reported high psychological distress among live-in caregivers, mainly related to concerns about family at home, an inability to fulfill requests for money, precarious employment and immigration, excessive work demands, lack of privacy, reproaches and abuse from employers, fear of deportation and job loss, feelings of alienation, extreme loneliness, and limited social support [14,15]. Based on caregivers’ reality of high stress and limited free time, it is imperative that they be provided with psychosocial support to reduce stress and promote their resilience.

Acceptance and Commitment Therapy (ACT) is an evidence-based psychological intervention that has proven to be effective in addressing mental and physical health challenges in both clinical and nonclinical populations [17-19]. Web-based ACT has also been found to be effective in addressing health challenges such as distress related to trauma, anxiety, depression, and chronic pain [20-22]. It promotes mental wellness and psychological flexibility by helping participants face their distress while engaging in the pursuit of valued actions such as self-care and other meaningful activities. ACT uses mindfulness-based exercises and experiential exercises to decrease experiential avoidance and to enhance one’s psychological flexibility through advancing six core processes: defusion (observing thoughts as thoughts), acceptance (of experiences of emotions and feelings), contact with the present moment (mindfulness), self-as-context (awareness and self-perspective), values (being clear about what matters), and committed action (based on values) [23,24].

The six ACT core processes can support migrant live-in caregivers in coping effectively with their mental health stress by enabling them to recognize their internal psychological struggles (eg, fusion with ideas and thoughts, avoidance of emotions and feelings) and apply ACT strategies like mindfulness, defusion from unworkable ideas, and engaging in committed action consistent with the value of self-care (eg, engaging in culturally syntonic activities like singing or praying and building social support networks). Given that live-in caregivers work long hours, have extremely limited free time, and lack social support, a web-based intervention offers flexible access while promoting virtual social connection.

This paper presents the study protocol of Women Empowerment - Caregiver Acceptance & Resilience E-Learning (WE2CARE), an internet-based ACT for promoting the mental well-being of migrant live-in caregivers. The study aims to reduce psychological distress (depression, anxiety, and stress) and promote committed actions of self-care. The study protocol received ethical approval from the research ethics review boards of Ryerson University (REB 2019-036) and the University of Toronto (RIS37623).

## Methods

### Study Design

A pilot community-based mixed methods design is used to assess the feasibility and effectiveness of the WE2CARE program. WE2CARE involves two sequential phases: a quantitative component that includes a waitlist randomized controlled trial and a qualitative component that involves focus groups. The waitlist control group will be used as an untreated comparison for the active experimental group to determine if the program had an effect.

### Participants and Recruitment Strategies

A total of 36 participants will be recruited by two community champions (ie, trusted members of the caregivers’ community collaborating with community health organizations serving the TFW population) and a snowball sampling technique. The inclusion criteria will include the following: (1) female adults aged ≥18 years, (2) residing in the Greater Toronto Area, (3) working on a temporary work permit as live-in caregivers, (4) able to speak and read English, (5) have internet access, and (6) able to take part in the 6-week intervention. They will be randomized to either the intervention arm or control arm using a random number generator.

In Phase 2 of our study, we will conduct two focus groups with a subset of participants from Phase 1 who indicate interest in participating. The aim of the focus groups is to gain a deeper understanding about participants’ views of the effectiveness of WE2CARE in promoting mental health, their perceived barriers and challenges in accessing this method of delivery (ie, online), and their recommendations to improve the content and method of delivery of this program.

### Treatment Intervention

#### Overview

The WE2CARE intervention consist of six e-learning modules that cover the ACT processes. Each week, participants are invited to complete an online, self-directed, interactive experiential session on ACT strategies (approximately one hour to complete) and to attend a 90-minute live online videoconference during which group discussions and question-and-answer sessions are facilitated by our research team members to support participants’ ACT practice.

#### Module 1: Reflecting on the Present Journey

This module provides the following: (1) an introductory overview of the course, (2) a guided interactive exercise for participants to reflect on their identity and experience as live-in caregivers, including their current distress and challenges, and the factors that motivate them to continue with their jobs (ie, both the immediate tangible rewards and internal values, such as caring for their families back home), and (3) an introduction to mindfulness as an approach toward all experiences, including psychological suffering.

#### Module 2: Developing Adaptive ACT Strategies to Deal With Distressing Experiences

This module supports participants to achieve the following: (1) recognize all thoughts as thought processes rather than being trapped by their literal content; for example, rather than worrying or arguing back with a thought about one’s uncertain future (“I am going to lose my job soon,” “my life will always be hopeless,” etc), one can mindfully recognize and accept them as thoughts and not reality, and focus on experiencing the here and now (defusion, acceptance, present moment); (2) overcome the tyranny of unhelpful rules or stories that constrict adaptive or valued behaviors (eg, “showing emotions is a weakness,” “I am no longer worthy,” “my needs are not important”) (defusion, acceptance); and (3) cultivate appreciation and gratitude, enabling them to connect to any positive aspects of their experience, however small, that may have been neglected due to fusion with the everyday challenges caused by difficult work conditions (eg, enjoying the sunrise, a cup of tea, a warm bath, fresh air; acceptance, present moment).

#### Module 3: Experiencing the Transcendent Self

This module engages participants in experiential exercises that support them to do the following: (1) reconnect with their former sense of self and identities before becoming a migrant caregiver, including their personal and cultural strengths; (2) defuse from any rigid conceptualized self (ie, being just an objectified migrant caregiver) and become aware through the “observer self” that they are not defined by their memories, their experiences, or labels imposed on them (rather, they are the holders of their lived experiences that continue to change throughout life); and (3) rediscover their strengths, passion, interests, and aspirations, and recognize their capacity to make choices based their values (eg, singing in a choir, attending a community event, choosing a new style of clothing; self-as-context, acceptance, present moment, values).

#### Module 4: Getting in Touch With Values and Meaning

This module engages participants to do the following: (1) explore their values in all domains in their life (self-care, family, friends, career, community, spirituality, etc); (2) evaluate whether their actions are moving toward or away from their values and supporting them to achieve balanced living; and (3) commit to self-care and other actions based on their values (values, committed action, acceptance).

#### Module 5: Building a Supportive Network

This module support participants to accomplish the following: (1) negotiate barriers to self-care by drawing from learned ACT strategies; (2) identify personal and community resources and reconnect with existing supports (eg, church, community associations); and (3) build new supports, such as mutual virtual support networks (values, defusion, acceptance, present moment, committed action).

#### Module 6: Committing to Valued Action

This module supports participants to consolidate their commitment to self-care by doing the following: (1) developing concrete short, intermediate, and long-term plans for self-care and (2) anticipating future barriers and formulating mitigating ACT strategies to sustain a commitment to self-care (committed action, values, present moment).

One of the therapeutic goals of WE2CARE is to motivate participants to engage collectively in building social support networks. The weekly videoconference, which will be cofacilitated by two trained research team members, will provide a viable venue to facilitate and model the development of a virtual community of mutual support, which may continue beyond the project.

A previous ACT intervention study conducted by our team members demonstrated that a participant-driven social support e-network (a closed group on Facebook) was effective in reducing social isolation, promoting community engagement, and strengthening mutual support.

### Data Collection

Informed consent will be obtained prior to data collection. Data will be gathered using both quantitative and qualitative tools, which include self-completed questionnaires and focus groups.

Quantitative data will be captured for participants in the control and intervention groups through self-report instruments that will be administered online pre- and postintervention. The intervention group will also complete the questionnaires 6 weeks postintervention. The survey questionnaires will include the following:

1. The preintervention survey will include sociodemographic and health-related questions.

2. The standardized scales administered preintervention, postintervention, and at the 6-week follow-up will include the following:

- The Depression, Anxiety and Stress Scale (DASS-21), a set of three self-report scales (21 items) designed to measure the emotional states of depression, anxiety, and stress.
- The Acceptance and Action Questionnaire (AAQ-2), a 7-item scale specifically designed to measure the impact of ACT core processes conceptualized as psychological flexibility.
- The Cognitive and Affective Mindfulness Scale – Revised (CAMS-R), a 12-item measure designed to capture a broad conceptualization of mindfulness not specific to any particular type of meditation training.
- The Multi-System Model of Resilience Inventory (MSMR-I), which consists of three subscales: internal resilience, pursuits and coping, and external resilience. Each subscale contains 9 self-reported items and indicates where the barriers to one’s resilience lie. These scales have shown good psychometric properties including internal consistency, test-retest reliability, and validity.

3. Participants’ evaluation of the WE2CARE program. Participants’ feedback about the program will be captured through a 12-item questionnaire that elicited their perceptions of the impact the program had on their mental and social well-being and their knowledge of how to access mental health services.

Qualitative data will be obtained through focus groups and interactive entries embedded throughout the 6 online modules, including questions on caregiving burden and factors that promote resilience in Module 1 and weekly valued action data logs on self-care in Modules 2-6.

### Data Analysis Planning

Descriptive statistics will be used to summarize participants’ sociodemographics, self-reported health characteristics, and practices. Both independent and dependent *t* tests will be performed to assess the mean score differences for each outcome measure across and within groups. An independent *t* test will be used to compare mean scores across the intervention and control groups. Paired *t* tests will be used to compare the following: (1) baseline and postintervention outcomes and (2) baseline and 6-week postintervention outcomes among intervention participants.

The number of intervention participants at low risk and at risk for anxiety, depression, and stress will also be compared at baseline, postintervention, and 6 weeks postintervention. The Fisher exact test will be used to determine if the number of intervention participants at risk for anxiety, depression, and stress changed across time. Risk for anxiety, depression, and stress will be assessed using the DASS-21 and AAQ-7. The DASS-21 contains three subscales that assess anxiety, depression, and stress. Those that score at a moderate severity level or above for anxiety, depression, and stress will be considered at risk. The cutoffs for a moderate severity level will be set at 9 for anxiety, 13 for depression, and 18 for stress. Based on the AAQ-7 scoring instructions, a score above 23 is associated with depression or anxiety symptoms.

Linear mixed effects models will be created for each outcome of interest from baseline to postintervention among participants in the intervention and control groups. Each model will include two fixed effects parameters and two random effects parameters, one for the intercept and one for the outcome. The intercept fixed effect represents the typical initial score of an outcome and the outcome fixed effect represents the typical increase or decrease in score from baseline to postintervention.

Participants’ feedback on the 12-item questionnaire will be stratified by study arm allocation and an independent *t* test will be used to determine if there are differences in survey responses by study arm allocation.

Quantitative data will be analyzed using SPSS (version 27; IBM Corp) and R (version 1.2.5003; R Foundation for Statistical Computing) for Windows. A threshold of .05 will be used to determine the level of significance for all *P* values.

Qualitative data include transcripts of the audiotaped focus groups and interactive entries embedded throughout the 6 online modules. Both data-driven inductive and question-driven deductive approaches will be used to analyze the data [25]. Each transcript and responses to each module will be reviewed independently by at least two research team members to generate initial codes, which will be reviewed by all team members and developed into a codebook through discussion and consensus. The team will then organize the coded data into themes (common patterns) and illustrations (unique aspects of experience) for reporting [25]. Integrity of data interpretation will be maintained by following strategies of credibility (member check, peer debriefing) and auditability [26,27].

## Results

The WE2CARE study protocol has been approved by the research ethics boards of Ryerson University (REB 2019-036) and the University of Toronto (RIS37623). Data collection occurred between November 2019 and May 2020. Of 18 participants in the intervention group, one did not complete the baseline questionnaire, while 6 of 18 participants in the control group withdrew from the study due to other competing life priorities. The total number of participants who completed the study was 29, with 17 in the intervention group and 12 in the control group. Our intervention group was divided into two cohorts of 9 and 8 participants, respectively. This allowed more opportunity for participants to share their experiences, mental health stressors, and application of ACT strategies during weekly videoconferences. The control group received the intervention after the two intervention cohorts were completed. Data analysis is in progress and results will be published in 2021.

## Discussion

WE2CARE is among the first studies exploring the effectiveness of ACT in addressing mental health challenges among live-in caregivers. Although there are numerous studies on web-based ACT intervention, few use group videoconferencing to promote peer connection and mutual support. One of the key goals of WE2CARE is to motivate participants to engage collectively in building social support networks. The weekly videoconferences, facilitated by trained counsellors, provide a viable vehicle to facilitate the building of a virtual community of mutual support. The results of both quantitative and qualitative data will guide the adoption of this intervention to best meet the mental health needs of migrant live-in caregivers.

## Appendix

Multimedia Appendix 1 External peer review report.

## Abbreviations

AAQ-2: Acceptance and Action Questionnaire
ACT: Acceptance and Commitment Therapy
CAMS-R: Cognitive and Affective Mindfulness Scale – Revised
DASS-21: Depression, Anxiety and Stress Scale
MSMR-I: Multi-System Model of Resilience Inventory
TFW: temporary foreign worker
WE2CARE: Women Empowerment - Caregiver Acceptance & Resilience E-Learning
