# Supplementary material for: Implementation of an Internet-Based Acceptance and Commitment Therapy for Promoting Mental Health Among Migrant Live-in Caregivers in Canada: Protocol
Source: JMIR Res Protoc. 2021 Sep 13;10(9):e31211. doi: 10.2196/31211 (PMC8694237; doi:10.2196/31211)
Supplement: Multimedia Appendix 1 [file resprot_v10i9e31211_app1.pdf]

THE RBC IMMIGRANT, DIVERSITY AND INCLUSION PROJECT AT RYERSON UNIVERSITY

Name of applicant	Mandana Vahabi
Proposal title	WE-CARE: Women Empowerment - Caregiver Acceptance & Resilience E-Learning: Web-based Acceptance and Commitment Therapy for Stress Reduction Among Migrant Live-in Caregivers

Reviewers comments:

Reviewer 1:
Thank you for this compelling, timely and innovative project. This population is in urgent need of support and this protocol has the capacity to interrupt the isolation of live in caregivers in order to support them.

Reviewer 2:
Dear applicant,
Congratulations for your excellent proposal. I would like to recommend the development of a plan for knowledge translation within Ryerson scientific community as well as the inclusion of the training for health and social sciences students as knowledge brokers. Good luck!

Reviewer 3:

- A strong, clearly written literature review that is easy to understand
- It could be beneficial to state your research objectives earlier (you should not have to read for 1.5 pages before we know what you are trying to accomplish). Once they are eventually stated they are clear and compelling
- Proven track record of generating peer reviewed publications from a previous round of funding through RBC. This research will build off of that previous work
- Good job of explaining the origins of We-Care. The methodology is thorough and well thought out.
- The timeline is well thought out and detailed
- The potential impact of creating a virtual community of support is particularly powerful and of value to study participants
- Excellent plan to have a community-based advisory committee made up of former temporary former workers and service providers. This will help to ensure that the research design and implementation remains ethical. A great deal of thought has also been put into ensuring that results are disseminated in ways that will benefit the study participants, and population of concern. Could be more specific about which journal articles you intend to publish in.
- The research team is very experienced in carrying out this type of work and there is a strong team of collaborators that will assist in gaining access to the study population, overcoming barriers, and ensuring that their rights are protected.
- A very well thought out budget. My only suggestion would be to consider providing a high honorariums given the time commitment of the participants and/or perhaps an honorarium for each completed survey
